# Intestinal Iron Homeostasis and Colon Tumorigenesis

**DOI:** 10.3390/nu5072333

**Published:** 2013-06-28

**Authors:** Xiang Xue, Yatrik M. Shah

**Affiliations:** 1 Department of Molecular & Integrative Physiology, University of Michigan, Ann Arbor, MI 48109, USA; E-Mail: xxue@umich.edu; 2 Department of Internal Medicine, Division of Gastroenterology, University of Michigan, Ann Arbor, MI 48109, USA

**Keywords:** iron, colorectal cancer, divalent metal transporter-1 (DMT-1), hepcidin, HIF, ferroportin (FPN)

## Abstract

Colorectal cancer (CRC) is the third most common cause of cancer-related deaths in industrialized countries. Understanding the mechanisms of growth and progression of CRC is essential to improve treatment. Iron is an essential nutrient for cell growth. Iron overload caused by hereditary mutations or excess dietary iron uptake has been identified as a risk factor for CRC. Intestinal iron is tightly controlled by iron transporters that are responsible for iron uptake, distribution, and export. Dysregulation of intestinal iron transporters are observed in CRC and lead to iron accumulation in tumors. Intratumoral iron results in oxidative stress, lipid peroxidation, protein modification and DNA damage with consequent promotion of oncogene activation. In addition, excess iron in intestinal tumors may lead to increase in tumor-elicited inflammation and tumor growth. Limiting intratumoral iron through specifically chelating excess intestinal iron or modulating activities of iron transporter may be an attractive therapeutic target for CRC.

## 1. Introduction

Colorectal cancer (CRC) is the third most common cancer in both men and women, and is the second leading cause of cancer death in the United States [1]. Each year about 150,000 new cases will be diagnosed in the United States [1], in European countries it is significantly higher, with the expected cases to be over 400,000 per year [2,3]. Current treatments for CRC are effective at early stages of disease, however over 40% of patients at the time of diagnosis present with late stage CRC where standard therapies are not effective [4]. Understanding the precise mechanisms that are critical in the progression of colon carcinogenesis may provide better treatment strategies for CRC. The etiological factors and molecular mechanisms underlying the pathogenesis of CRC are multifactorial and complex [5]. In addition to inherited mutations and environmental interactions, dietary nutrients play a key role in the development of CRC [6].

## 2. Mechanism of Iron Absorption

**Figure 1:**
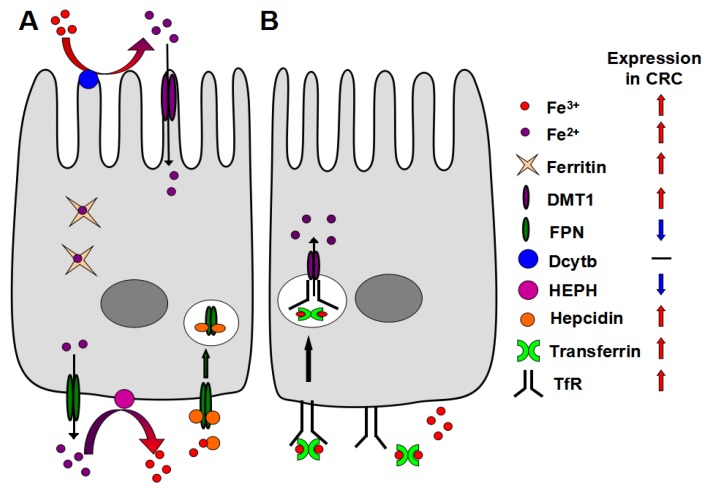
Mechanism of iron accumulation in colon tumors. (**A**) Apical increase in divalent metal transporter 1 (DMT-1) leads to increase in uptake of luminal iron in tumors. The iron is further sequestered in colon epithelial cells through a decrease in hephaestin (HEPH) and increase in hepcidin-mediated ferroportin (FPN) degradation; (**B**) An alternative or complimentary mechanism leading to increase of intratumoral iron is through the increase in transferrin-TfR mediated uptake of circulating iron.

Iron levels must be precisely controlled to meet systemic demands but avoiding deleterious iron overload [7]. A major node of regulation occurs at the proximal small intestine. Dietary iron absorption occurs mainly in the lumen of duodenum by a tightly regulated process. This process is influenced by the individual’s iron status and by several factors in the diet. Ascorbic acid enhances, whereas polyphenols and calcium inhibit iron absorption [8,9,10]. There are two major forms of nutritional iron, heme and non-heme. Iron acquired from meat is 40% heme iron and 60% non-heme iron. In addition, the iron acquired from fruits, vegetables, grains, and nuts is non-heme iron [11]. Heme is taken up from the gut lumen into intestinal epithelial cells as an intact metalloporphyrin. Currently the transporter responsible for heme uptake is unclear (proposed to be PCFT/HCP1, SLC46A1) [12,13]. After entering the enterocyte, heme is broken down by heme oxygenase into ferrous iron (Fe^2+^), bilirubin and carbon monoxide [14]. Dietary non-heme iron is in the ferric form (Fe^3+^) and is reduced to ferrous iron (Fe^2+^) by ferric reductases such as duodenal cytochrome b (Dcytb) [15], and then is transported by the divalent metal transporter 1 (DMT-1, also known as SLC11A2, NRAMP2 and DCT1) at the apical surface of enterocytes [16,17]. Dietary iron can be stored as ferritin within the enterocytes [18], or under increased systemic requirements of iron, Fe^2+^ is exported across the basolateral membrane of the enterocyte into the circulation by ferroportin (FPN, also known as IREG1) [19,20]. Upon release from FPN, Fe^2+^ is oxidized into Fe^3+^ by ferroxidase such as ceruloplasmin (Cp) and hephaestin (HEPH) [21,22,23], and rapidly binds to plasma transferrin [24] (Figure 1A). Transferrin-diferric iron complex is taken up into cells by binding with transferrin receptor (TfR) on the cell surface, followed by internalization through clathrin-mediated endocytosis [25,26] (Figure 1B). The efficiency of colonic iron absorption is only about 14% of duodenum, which is due to a lower expression of iron absorptive genes such as DMT1, TfR and ferritin in normal colon tissues than duodenum [27,28]. Interestingly, FPN is highly expressed in the colon, to the same extent as the duodenum [27]. The expression of these iron transporters is altered in CRC and is discussed in detail below. Intestinal iron absorption is mainly regulated by hypoxia-inducible factor (HIF)-2α, hepcidin, and iron regulatory proteins (IRPs) and their role in iron homeostasis has been reviewed in detail elsewhere [29,30,31,32,33,34]. In addition to local regulation of iron transporters by HIFs, hepcidin is an antimicrobial peptide that is synthesized in the liver and secreted into the circulation, binds to FPN on the basolateral membrane and leads to the internalization and degradation of the iron exporter [35,36,37].

## 3. Increased Systemic Iron Is a Risk Factor for CRC

As an indispensable nutrient for life, iron is essential for erythropoiesis and numerous other cellular processes including cell growth. Iron deficiency leads to anemia and affects millions of people worldwide. On the other hand, iron overload caused by hereditary mutations or excess dietary iron uptake has been identified as a risk factor for CRC through population and animal studies [38]. A retrospective literature review identified 26 individual reports that assessed the role of iron exposure in CRC risk [39]. Each report was graded and scored based on unbiased rigorous criteria to evaluate the validity of each report. From the twenty-six reports, eleven were given an acceptable quality rating. Nine of the eleven reports that were graded high demonstrated a positive correlation with increased CRC risk and iron exposure. A recent meta-analysis also supports a positive dose-response correlation of dietary iron intake with CRC risk [6]. However, to be pointed out, a nested-control study that measured iron status in women did not support the role of iron in the pathogenesis of CRC [40].

### 3.1. Hemochromatosis

Mutations in the hemochromatosis gene HFE can lead to hereditary hemochromatosis due to inappropriately high iron uptake from the diet resulting from decreased hepatic expression of the iron regulatory hormone hepcidin [41,42]. These individuals have an increased risk of developing CRC. Recent studies showed that the risk of developing CRC is doubled in patients with the two major HFE mutations, C282Y and H63D [43,44]. When these carriers consume high quantities of iron, the cancer risk is further increased [45], implying iron overload is a contributory factor to colon tumorigenesis.

### 3.2. Dietary Consumption

Red meat is a major component of diet in Western countries [11]. A recent meta-analysis indicated that red and processed meat intake is associated with increased risk of colorectal adenomas [46], which support that iron may play a key role in an early stage of the colorectal carcinogenesis. In large population-based studies, the CRC risk is positively associated with the amount of consumed red meat [47,48]. Increased risk of CRC in red meat consumers is entirely dependent on heme iron abundance in red meat and independent of dietary fat content or inorganic iron [48,49,50]. Feeding rats with heme leads to damage in the colonic mucosa surface and compensatory hyperplasia [50]. Down-regulation of inhibitors of proliferation such as Wnt inhibitory factor 1, Indian hedgehog and BMP2 are proposed as a mechanism for heme-induced colonic hyperplasia [51]. Furthermore, heme iron from red meat could also promote colon carcinogenesis through its catalytic effects on the formation of carcinogenic *N*-nitroso compounds or cytotoxic and genotoxic secondary lipid oxidation products, such as 4-hydroxy-2(*E*)-nonenal (HNE) [52,53,54]. Trapping heme with calcium phosphate or green vegetable derived chlorophyll could inhibit the cytolytic and hyperproliferative effects of dietary heme and reduce CRC risk in meat-eaters [49,55,56,57,58]. In addition, ferrous iron is widely supplemented in the American diet [39]. Supplemental iron is positively associated with distal colon cancer, which may cause enhanced free radical production in the colon [59,60].

### 3.3. Mouse Models of CRC

1,2-dimethylhydrazine (DMH) is a carcinogen that initiates colorectal tumorigenesis in rodent models. Chronic treatment of DMH up to 20-weeks will lead to a tumor response with a 100% incidence with multiple tumors in the distal colon [61]. In mice fed iron-rich diets, DMH-induced colon tumors were robustly increased with respect to tumor size and multiplicity [62]. Consistent with this data, iron enriched diets increased colon tumorigenesis in genetic models of colon cancer [63].

## 4. Low Iron Is Protective in CRC

Two separate studies using genetic mouse models of CRC demonstrated that mice on low-iron diet had decreased colon tumors compared to mice on an iron-replete diet [63,64]. A robust decrease in tumor cell proliferation was noted in both studies following low iron treatment. Assessing decreased iron stores in humans is very difficult since the major parameters to assess body iron store such as serum iron, hematocrit, transferrin saturation, and ferritin are not accurate or sensitive measures [65]. These parameters can display large variations in healthy populations. Moreover there are gender and age specific changes in several of these parameters. However, assessing the cancer risk in frequent blood donors who deplete iron stores through loss of blood compared to those who do not donate blood found a significant decrease in CRC risk [66]. More recently a multi-center single blinded clinical trial in which individuals were phlebotomized at six-month intervals for an accrual period of 3.5 years and a follow-up period of 2.5 years demonstrated that the phlebotomized group was associated with a lower risk of all cancers [67]. More specifically a significant decrease in CRC risk was demonstrated. Moreover, the phlebotomized group that did get cancer had a decreased mortality compared to the control group with cancer.

## 5. Altered Local Iron Homeostasis in CRC

The expression of proteins associated with cellular iron uptake machinery such as DMT-1 and TfR1 is up-regulated in CRC, whereas the expression of the iron export proteins such as FPN and HEPH is decreased in advanced CRC [38] (Figure 1). Consistent with this data, iron content is increased in human colorectal tumors and tumors from mouse models of CRC compared to normal adjacent tissues as demonstrated by Prussian blue staining [64,68]. Similar alterations that result in increased iron acquisition and decreased iron export by the tumor are also observed for other tumors such as breast [69,70]. This data demonstrate that local iron requirements are increased in tumors compared to normal tissues, and the increase in intratumoral iron may be critical in tumor growth.

### 5.1. DMT-1

DMT-1 is expressed at significant levels in the colon and is dramatically increased by iron-deficient diet [71]. DMT-1 is highly induced in colon adenomas and carcinomas compared with normal colon tissue isolated from patients [72,73,74]. Two independent groups have recently demonstrated that the expression of DMT-1 protein is increased in mouse intestinal tumors, and this increase is dependent on HIF signaling pathway and Wnt signaling pathway, respectively [63,64].

### 5.2. Transferrin and TfR

A large cohort study found that high transferrin saturation level is associated with excess risk for CRC [75]. In colon tissue, TfR1 is expressed in normal colonocytes [22], and further increased in CRC of Dukes A or B grade [76]. The expression level of TfR1 regulates cellular iron homeostasis and is correlated with the rate of cell proliferation [77]. TfR1 is directly regulated by hypoxia signaling and c-myc [77,78,79]. TfR1 overexpression in human tumors confers a growth advantage to cells and greatly enhances the rate of c-myc-mediated tumorigenesis, whereas its inhibition greatly decreases cellular proliferation and results in G1 arrest [80]. Increased TfR1 tumor cell surface expression is correlated to increased malignancy and thus may provide prognostic information for malignant tumors [81,82]. A second transferrin receptor TfR2 was recently cloned [83] that plays a key role in the regulation of iron homeostasis [84]. TfR2 is frequently expressed in various human cancer cell lines. In primary colon tumors, TfR2 was expressed in about 26% of CRC patients [85]. The expression of TfR2, together with increased TfR1, may represent a mechanism by which CRC cells accumulate iron.

### 5.3. Ferritin

Ferritin is a protein that regulates intracellular iron homeostasis by sequestering it within a hollow heteromultimer of heavy (H-ferritin) and light subunits (L-ferritin) [86]. Its ability to sequester the nutrient gives ferritin the dual functions of iron storage and iron detoxification [87]. As an indicator of iron storage, serum ferritin has a positive association with the formation of colorectal adenomatous polyps [88,89,90]. The elevated serum ferritin may be in part derived from the elevated ferritin content of colon neoplasms [91]. At the same time, as an intracellular iron scavenger, CRC cells enriched in endogenous ferritin are resistant to oxidant-producing chemotherapeutic agents, and prolonged iron exposure further increases the overall content of ferritin in these cells [92]. On the contrary, the down-regulation of H-ferritin by the proto-oncogene c-myc in cells can increase the availability of intracellular free iron, leading to oxidative stress, cell transformation and cell proliferation [93]. These data demonstrate a complex and biphasic role of ferritin in CRC, where in context-dependent manner low and high ferritin may be advantageous to tumor growth.

### 5.4. HEPH

HEPH is expressed at high levels throughout the small intestine and colon [94]. HEPH is critical for oxidation of Fe^2+^ to Fe^3+^ allowing efficient loading of diferric iron onto transferrin. Although no studies have directly assessed HEPH expression in CRC, mining publicly available CRC microarray datasets [95] found that HEPH was significantly decreased in CRC from three independent studies [96,97]. Moreover, HEPH is directly regulated by the homeobox transcription factor CDX2. In CRC cells, overexpression of CDX2 activation rapidly increases the expression of HEPH and results in suppressed intracellular iron, whereas CDX2 inhibition leads to lower HEPH expression. CDX2 and HEPH expression are well correlated in normal and tumor tissues [98], and CDX2 expression is downregulated in CRC.

### 5.5. Hepcidin and FPN

Hepcidin is not expressed in normal colon epithelium, but it is increased in CRC tissue with a repression of FPN [99]. Furthermore, urinary hepcidin expression is positively associated with increasing T-stage of CRC. Thus, increases in both tumor-produced and systemic hepcidin could lead to decreased tumor FPN levels and greater iron retention by the tumor.

## 6. Oxidative Stress and CRC

**Figure 2:**
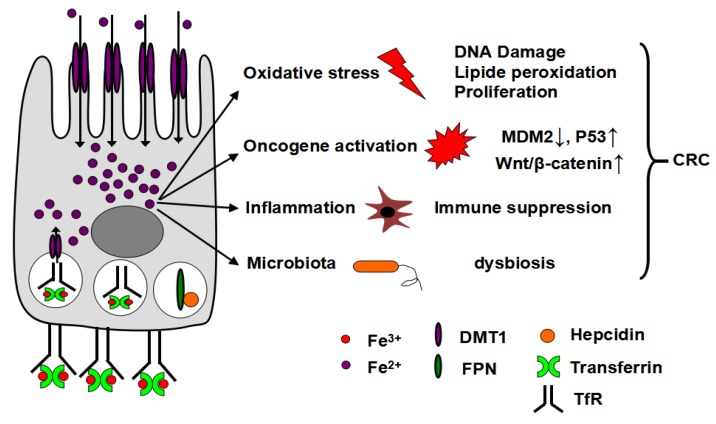
Iron-mediated colorectal cancer (CRC) progression. Through increased iron acquisition by DMT-1 from the diet or holo-Tf-TfR1 from the circulation, and decreased iron export by hepcidin-mediated degradation of FPN or decreased FPN expression, iron accumulation in colon tumors leads to an increase in reactive oxygen species (ROS) production, oncogene activation, pro-inflammatory mediators and dysbosis which contribute to an increase in the growth of CRC.

Iron is essential for all living organisms and serves as a cofactor for many proteins and enzymes participating in numerous biological and cellular processes such as energy metabolism, cellular respiration, oxygen transport, DNA synthesis and cell growth [100,101]. In addition, iron accumulation leads to a robust increase in reactive oxygen species (ROS) formation, which is a critical mechanism leading to increase in colon tumorigenesis. Increased cellular iron can lead to the formation of ROS through the Fenton and Haber-Weiss reaction by catalyzing the decomposition of hydrogen peroxide [102]. Increased oxidative stress is genotoxic and can lead to DNA damage and subsequent gene mutations important in the initiation of colon tumorigenesis [103]. Iron is a potent genotoxic mutagen in primary non-transformed colon cells, preneoplastic colon adenoma cells and human CRC cell lines [104,105,106]. Moreover oxidative stress can lead to lipid peroxidation, which is enhanced in colon tumors compared to normal colonic mucosa [107]. High dietary iron increases lipid peroxidation in the colon from both mice and rats [108,109,110]. Lipid peroxidation propagates the free radical reactions and facilitates the formation of DNA-adducts that may contribute to tumorigenesis [111]. Lastly, oxidative stress induces a pleiotropic effect in cancers and can regulate proliferation, apoptosis, mitochondrial metabolism, and inflammation (Figure 2). The involvement of iron-induced oxidative damage in CRC is further demonstrated by the fact that the antioxidant *N*-acetylcysteine (NAC) inhibits dietary iron-induced CRC progression and nitrotyrosine-positive cell number in a colitis-associated CRC model [112,113].

## 7. Additional Pathways of Iron Induced-Tumorigenesis

### 7.1. Oncogene Activation

Colorectal carcinogenesis is a multi-step process involving the formation of adenomatous polyps and their subsequent progression to malignancy. At the molecular level, this process is reflected by sequential events of gene mutation and activation of important molecular pathways [114]. Here we will discuss two key activated signaling pathways critical in CRC (Figure 2): (i) The ubiquitination and degradation of the tumor suppressor gene p53; and (ii) Wnt/β-catenin signaling. Iron downregulates whereas iron chelator deferoxamine (DFO) upregulates the expression of mouse double minute gene 2 (MDM2), which is a ubiquitin ligase involved in the degradation of the p53 [115]. Iron overload-mediated reduction in MDM2 levels leads to p53 upregulation and consequent induction of antioxidant enzymes, thereby providing a mechanism to counteract iron-related oxidative stress. On the contrary, DFO causing moderate iron deficiency increases MDM2 level and consequently decreases p53 levels. Interestingly, DFO at toxic doses can also increase nuclear protein level and DNA-binding activity of p53, which causes the cell cycle arrest effects and anti-tumor activities [116,117]. Aberrant activation of Wnt/β-catenin signaling contributes to the development of CRC [118]. The adenomatous polyposis coli (*Apc*) gene, a key Wnt signaling factor for the regulation of mucosal epithelial maturation, is mutated in a majority of patients with familial, sporadic, and colitis-associated CRC [119,120,121]. APC mutations lead to nuclear accumulation of β-catenin and constitutive activation of the transcription factor T-cell factor/lymphoid enhancer factor (TCF/LEF), and results in activation of target genes, including cyclin D1 and c-myc, both of which are positive regulators of cell proliferation [122,123]. Iron can increase Wnt signaling following the loss of APC function [124], whereas iron chelators can block Wnt signaling and decrease the expression of cyclin D1 and c-myc to suppress cell growth [101,125,126].

### 7.2. Tumor Inflammatory Response

Chronic inflammation is a major hallmark of CRC [127]. The relative risk for developing CRC in inflammatory bowel disease (IBD) patients is 11 times higher than that of the general population [128]. Moreover, sporadic colon tumors have a heightened inflammatory response without any preceding chronic inflammatory condition. The increase in pro-inflammatory response in tumors in general is thought to function as a growth supportive mechanism. Consistent with this notion there is strong evidence that anti-inflammatory drugs inhibit colon tumorigenesis, reduce the risk of CRC, and prevent the recurrence of adenomatous polyps [129]. In mouse models, dietary iron supplementation worsens dextran sodium sulfate-induced chronic inflammation and increases colorectal tumorigenesis, suggesting a tumor-promoting role of iron in the presence of inflammation [112]. The inflammatory microenvironment in tumors is highly complex, and not all tumoral inflammatory responses promote growth. The tumor immune response can also eliminate transformed cells. Currently, mechanisms that regulate the pro-tumorigenic *versus* the anti-tumorigenic balance of the tumor immune response are unclear. However, it is thought that immune suppression of effector cells is important in tipping the balance by which the tumor microenvironment promotes growth (Figure 2). A proposed mechanism by which iron in the tumor epithelium can result in immunosuppression is quenching the nitric oxide-mediated activation of NK cells [130]. Moreover, increased iron in macrophages leads to an attenuated inflammatory response by decreasing pro-inflammatory mediator production from macrophages [131,132,133].

### 7.3. Iron and Microbiota

The last 10 years has demonstrated the importance of the gut microbiota to health and disease. The intestinal lumen bacterial content is estimated to be an order of magnitude higher of number of cells than all of our somatic cells. The intestinal lumen is the most densely occupied microbial ecosystem found to date. Gnotobiotic mouse models and broad-spectrum antibiotic gut sterilization experiments have demonstrated the importance of the gut microbiota in CRC [134,135,136,137,138]. Accumulating evidence suggest that microbial dysbiosis is associated with the initiation and progression of CRC through disturbing mucosal immune response between host and microbiota [139,140,141,142] (Figure 2). The role of intestinal iron accumulation in gut dysbiosis and its implication in CRC has not been directly tested. However, several studies on rodent models and in humans have demonstrated that changes in dietary iron intake and luminal iron can have a profound influence in the gut microbiota [143,144,145,146,147,148,149]. Moreover, it has been hypothesized that the increase in CRC incidence in African American compared to native Africans is due to different compositions of intestinal microbiota between these two populations. A higher colonic population of potentially toxic hydrogen and secondary bile-salt-producing bacteria is observed in African Americans [150].

## 8. Conclusions

Systemic iron levels and local increase in iron accumulation in tumors significantly impacts the progression of CRC. Currently much more is known about modulating systemic iron levels and its role in CRC progression compared to intratumoral iron. Iron chelation therapy is efficacious in both animal studies and clinic settings. Furthermore, anti-TfR1 antibodies exhibit antitumor activity through inhibiting iron uptake. However, systemic decrease of iron can exacerbate anemia that is present in most patients with CRC. The higher iron utilization by cancer cells than normal cells provides a rationale for reducing iron content in the colon to limit the progression of colon tumors. Further studies are required to understand the precise mechanism by which colon tumors accumulate iron and its role in CRC progression. DMT1 and TfR1 are both up-regulated in CRC, however no mechanistic study has been performed to understand if either DMT1 or TfR1 is essential for iron accumulation in CRC and its progression. These studies are complicated by the fact that disruption of their function in the intestine will cause systemic iron deficiency in mouse models. The future focus of iron restriction therapies in CRC will have to be on mechanisms that can specifically restrict iron in the colon, and the development of pharmacological methods to modulate colon iron could have the potential to impact human CRC.
